# Tailoring the composition of biocopolyester blends for dimensionally accurate extrusion-based printing, annealing and steam sterilization

**DOI:** 10.1038/s41598-022-24991-z

**Published:** 2022-11-25

**Authors:** F. Burkhardt, V. D. Schmidt, C. Wesemann, C. G. Schirmeister, S. Rothlauf, S. Pieralli, L. S. Brandenburg, L. Kleinvogel, K. Vach, B. C. Spies

**Affiliations:** 1 Department of Prosthetic Dentistry, Medical Center—University of Freiburg, Center for Dental Medicine, Faculty of Medicine, University of Freiburg, Hugstetter Str. 55, 79106 Freiburg, Germany; 2 Freiburg Materials Research Center FMF and Institute for Macromolecular Chemistry, Albert-Ludwigs-University Freiburg, Stefan-Meier-Str. 21, 79104 Freiburg, Germany; 3 Basell Sales & Marketing B.V., LyondellBasell Industries, Industriepark Höchst, 65926 Frankfurt a.M, Germany; 4 Department of Oral and Maxillofacial Surgery, Medical Center—University of Freiburg, Center for Dental Medicine, Hugstetterstr. 55, 79106 Freiburg, Germany; 5 Medical Center—University of Freiburg, Institute for Medical Biometry and Statistics, Faculty of Medicine, University of Freiburg, Stefan-Meier-Str. 26, 79104 Freiburg, Germany

**Keywords:** Biomaterials, Materials for devices, Characterization and analytical techniques

## Abstract

Fused filament fabrication (FFF) represents a straightforward additive manufacturing technique applied in the medical sector for personalized patient treatment. However, frequently processed biopolymers lack sufficient thermal stability to be used as auxiliary devices such as surgical guides. The aim of this study was to evaluate the dimensional accuracy of experimental biocopolyester blends with improved thermal characteristics after printing, annealing and sterilization. A total of 160 square specimens and 40 surgical guides for oral implant placement were printed. One subgroup of each material (n = 10) underwent thermal annealing before both subgroups were subjected to steam sterilization (134 °C; 5 min). Specimens were digitized and the deviation from the original file was calculated. The thermal behavior was analyzed using differential scanning calorimetry and thermogravimetric analysis. A one-way ANOVA and t-tests were applied for statistical analyses (p < 0.05). All biocopolyester blends showed warpage during steam sterilization. However, the material modification with mineral fillers (21–32 wt%) and nucleating agents in combination with thermal annealing showed a significantly reduced warpage of printed square specimens. Geometry of the printing object seemed to affect dimensional accuracy, as printed surgical guides showed less distortion between the groups. In summary, biocopolyesters did benefit from fillers and annealing to improve their dimensional stability.

## Introduction

Additive manufacturing (AM) processes such as stereolithography (SLA) and digital light processing (DLP) are increasingly applied in the dental field. One example is the customized production of surgical guides for oral implant placement^1^. These vat polymerization processes include the curing of liquid photopolymer resins with ultraviolet light, which enables the precise production of the final objects^2^. However, the printed objects require extensive post-processing in the form of rinsing, washing, and light-curing^3^ and an elution of monomers in the moist oral environment may occur^4^. The entire manufacturing process requires experienced personnel and the equipment and materials are cost intensive.

In contrast, fused filament fabrication (FFF), also known as fused deposition modeling (FDM), represents a cost-effective and straightforward AM method^5^. A thermoplastic material is fed into a heated nozzle, where it is molten and subsequently deposited layer-wise until the final object is built. The finished parts do not require further cleaning or light curing steps. The applied materials are in need of a low warpage tendency, a fast and stable fusion of the deposited layers, and good adhesion to the build platform^6,7^.

Thermoplastic materials used for FFF differ in their amorphous or semi-crystalline nature^8^. In the solid state below the glass transition temperature, they are present in a glass phase and thus exhibit brittle and hard properties. The amorphous polymers do not show a specific melting point, but transform into a viscous state when the glass transition temperature is exceeded. Viscosity decreases over a wide temperature range, allowing for straightforward processing. However, the difference between processing temperature and glass transition temperature in semi-crystalline polymers is relatively high, which leads to more difficulties in printing^9^.

Shrinkage occurs during the cooling process, particularly due to crystallization^10,11^. Warpage results from shrinkage stress that arises when a layer is cooled over an already cooled layer. The larger the temperature difference between the two layers, the higher the warpage. Polymers with an increased crystalline content are therefore more prone to shrinkage and warpage^12^. Thus, mainly amorphous polymeric acrylonitrile–butadiene–styrene copolymers (ABS) or polycarbonates (PC) are used for extrusion-based printing. Moreover, semi-crystalline thermoplastics with a low crystallinity and a low crystallization rate, such as polylactic acids (PLA) or polyhydroxyalkanoates (PHA), are widely used^2,13–15^. PLA and PHA belong to the biopolymers, which offer a broad source of raw material, making them available at low cost^16^. They are biodegradable under certain conditions and might represent a sustainable alternative for single-use auxiliary products such as surgical guides for oral implant placement^17^. However, due to their low crystallinity, biopolymers do not exhibit the same mechanical properties and chemical resistance as polymers with a higher crystallinity. Thermal annealing of PLA after extrusion based printing can lead to an increased crystallinity and consequently enhanced mechanical strength^18^. In addition, the mechanical and thermal properties can be optimized by fillers such as silica or natural fibers^9,19^. With regard of a medical application of FFF-printed parts such as surgical guides or medical instruments in a sterile environment, little is known concerning the effect of steam sterilization on the dimensional accuracy and crystallinity of extrusion-based printed biopolymers. High dimensional accuracy of extrusion-based printed parts such as surgical guides after steam sterilization is crucial for the use in a medical environment.

Therefore, this study aimed to evaluate different experimental biocopolyester blends for medical extrusion-based printing. The influence of different material modifications, thermal annealing, and different printing parameters on the dimensional accuracy were investigated. The crystallization behavior was observed by differential scanning calorimetry (DSC) while thermal stability of the biocopolyester blends and the inorganic filler content were studied using thermogravimetric analysis (TGA) to determine a correlation of filler content and dimensional stability.

## Materials and methods

### Evaluated materials

In the present study, four experimental biocopolyester blends (A, B, C, D) were investigated in comparison to a market-available biodegradable (DIN EN ISO 14855) reference material (R, GreenTec Pro, Extrudr, Lauterbach, Austria). According to the manufacturer, all of the evaluated biocopolyester blends (Extrudr) and the reference polymer were based on the biopolymers poly lactide acid (PLA), polybutylene adipate terephthalate (PBAT), and polyhydroxyalkanoate (PHA). The reference material R has a melting temperature > 190 °C, and a Vicat softening temperature (VST) of 160 °C. Polymers A, B, and C have the same basic composition, with A and B additionally containing melt stabilizers, nucleating agents and inorganic fillers, such as calcium carbonate. According to the manufacturer, materials C and D, and reference material R have a low filler content, however, the exact filler content was not available. The manufacturer does not provide the exact material composition.

### Experimental setting

For each group, n = 20 rectangular specimens were manufactured by extrusion-based printing. Ten samples per group were printed in one batch and thermally annealed (At, Bt, Ct, Dt, Rt). The second batch containing the other ten samples did not undergo thermal post-processing (A0, B0, C0, D0, R0). This resulted in ten subgroups, which were subjected to a steam sterilization procedure. To determine their dimensional accuracy, all specimens were measured after 3D printing, annealing, and sterilization. The crystallization behavior was observed by differential scanning calorimetry (DSC) and the relationship between dimensional stability and fillers was studied using thermogravimetric analysis (TGA). In order to evaluate the influence of different printing parameters, additionally 60 square test specimens for group At were printed to investigate the influence of printing temperature, infill content, and number of outer shells (At with modified printing parameters; group Atm). Based on the results of the square specimens, surgical guides for implant placement were printed for the groups At, Bt, Rt and Atm (n = 10 per group) and their dimensional accuracy was assessed after steam sterilization.

### 3D printing of test specimens

**Figure 1:**
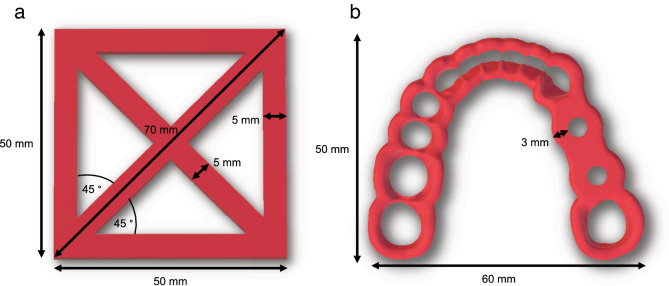
Scheme of the investigated square test specimen (**a**) and of the surgical guide for the placement of oral implants (**b**).

A rectangular square with a side length of 50 mm and a height of 0.3 mm, which contained two diagonal struts between the opposite corners, was chosen as geometry for the test specimens based on a previous study^20^ (Fig. 1a). In addition, a surgical guide used for the insertion of oral implants was selected as a second, practice-related geometry (Fig. 1b). The CAD files of the square specimens were created with the software Meshmixer (Autodesk, Mill Valley, CA, USA). The file of the surgical guide was generated with the implant planning software CoDiagnostix (Dental Wings GmbH, Chemnitz, Germany) and virtually trimmed with the software Meshmixer in the occlusal region to leave a marginal scaffold around the supporting teeth. The standard tessellation language (STL) files of the square specimens and of the surgical guide were transferred to the slicer software (PrusaSlicer, Prusa, Prague, Czech Republic). All of the test specimens were additively manufactured in the XY-plane using an extrusion-based desktop printer (i3 MK3, Prusa) with a steel nozzle (0.4 mm). Support structures were only used in the region of the guide sleeve of the surgical guide. Ten specimens were manufactured simultaneously on the build platform. The following standard settings were applied: nozzle temperature of 215 °C, temperature of the printing bed of 60 °C, acceleration of 1000 mm/s^2^, and layer height of 0.15 mm. The infill was 100% (linear infill pattern) and three horizontal and vertical outer shells were applied (Table 1). For further evaluation of the various printing parameters, the group At samples were also printed with a nozzle temperature of 230 °C, and a first layer of 230 °C followed by 200 °C. In addition, the influence of 50% infill (gyroid pattern) and printing without infill (hollow) was investigated. Additionally, the influence on the dimensional accuracy of 1 and 4 outer shells was evaluated. The surgical guides of the Atm group were printed with the following modified parameters: first layer with a nozzle temperature of 230 °C followed by 200 °C, no infill and 4 outer shells.

**Table 1:** Standard printing parameters and modified parameters.

Settings	Standard	Modification 1	Modification 2	Modification 3	Modification 4	Modification 5	Modification 6
Nozzle temp	215 °C	230 °C	230 °C / 200 °C	215 °C	215 °C	215 °C	215 °C
Printing bed temp	60 °C	60 °C	60 °C	60 °C	60 °C	60 °C	60 °C
Acceleration	1000 m/s^2^	1000 m/s^2^	1000 m/s^2^	1000 m/s^2^	1000 m/s^2^	1000 m/s^2^	1000 m/s^2^
Layer height	0.15 mm	0.15 mm	0.15 mm	0.15 mm	0.15 mm	0.15 mm	0.15 mm
Infill	100%	100%	100%	50%	0%	100%	100%
Shells	3	3	3	3	3	1	4

### Thermal annealing

The thermal annealing for the groups At, Bt, Ct, Dt, Rt, and Atm was performed in a preheating oven (KMP 6, MIHM-VOGT, Stutensee-Blankenloch, Germany). The test specimens were first heated to a temperature of 140 °C for 30 min. Thereafter, the temperature was maintained for a period of 90 min before the test specimens were cooled to room temperature over a period of 30 min.

### Sterilization

All of the test specimens were steam sterilized in an autoclave (Webeco, Series EC, Selmsdorf, Germany) at 134 °C for 5 min (3114 mbar). The total running time of the processing was 57 min.

### Assessment of dimensional accuracy

**Figure 2:**
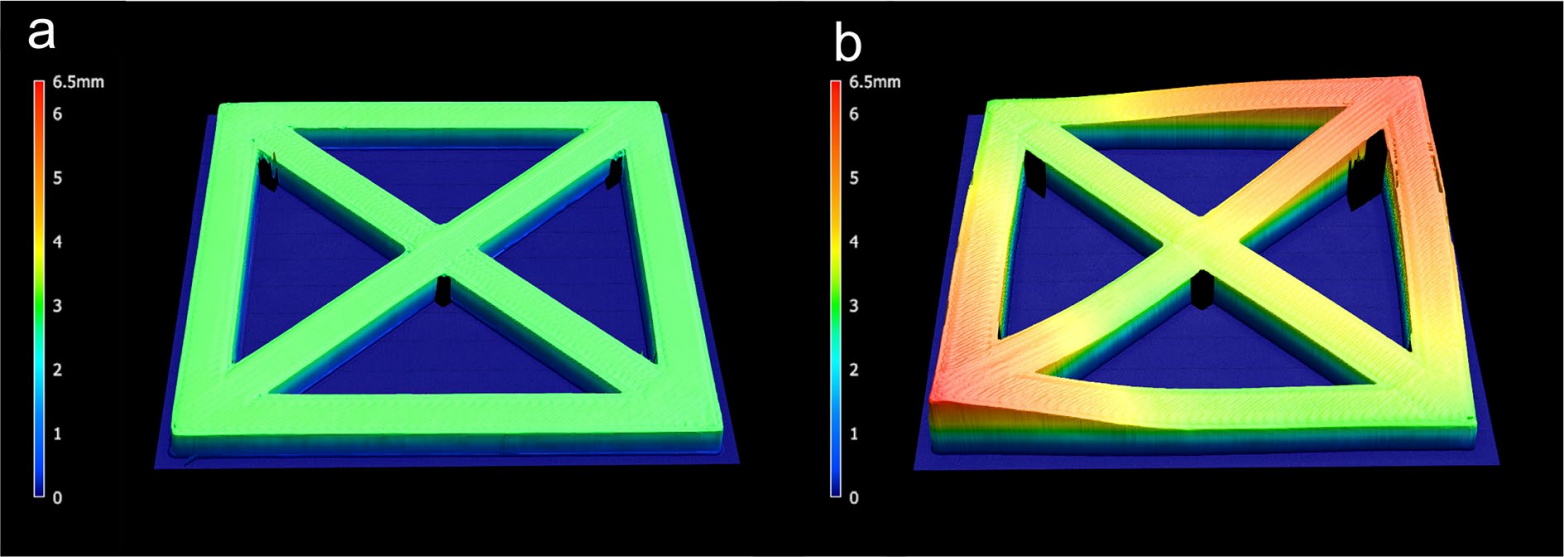
Scan of a test specimen (group R) after 3D printing (**a**) and after steam sterilization (**b**). The color map indicates the height of the specimen. The volume of the original STL file was subtracted from the volume of the scans to determine the respective deviation.

The dimensional accuracy of the square specimens was determined after 3D printing, annealing, and steam sterilization. Therefore, all printed square test specimens were digitized with a 3D profilometer (VR-500, Keyence, Osaka, Japan). The measured value describes the volume of the specimen and the enclosed volume underneath it. To obtain solely the resulting deviation, the volume of the original geometry STL file was subtracted from the measured volume (Fig. 2). The printed surgical guides were scanned with a 3D coordinate measuring machine (VL-500, Keyence). The scans of the surgical guides were imported into an inspection software (Geomagic Control X, 3D Systems, Rock Hill, USA) and compared to the original STL using a local best-fit algorithm according to Gauss. The mean deviation was calculated according to the root mean square (RMS) value, and the orientation of the deviation was evaluated according to the negative and positive mean values. Heatmaps indicating the positive and negative deviations were created for visualization.

### Differential scanning calorimetry (DSC) and Thermogravimetric analyses (TGA)

Thermal characterization was conducted through DSC using a DSC 6200 F1 (Seiko, Chiba, Japan) at a heating rate of 10 K min^−1^. In addition, TGA was performed on a STA 409 (Netzsch, Selb, Germany) within a temperature range of 50–650 °C at a heating rate of 10 K min^−1^ in air.

### Statistical analysis

For descriptive analyses, the mean, median and standard deviation were computed. A one-way ANOVA was used to examine differences among the groups. To correct for the multiple testing problem, the results of pairwise comparisons were adjusted by the Student–Newman–Keuls's method. For comparisons of time differences within each material the paired t-Test was applied. The calculations were performed with the statistical software STATA 17.0 (StataCorp, College Station, Texas, USA).

## Results

### Assessment of dimensional accuracy after annealing and after steam sterilization

**Figure 3:**
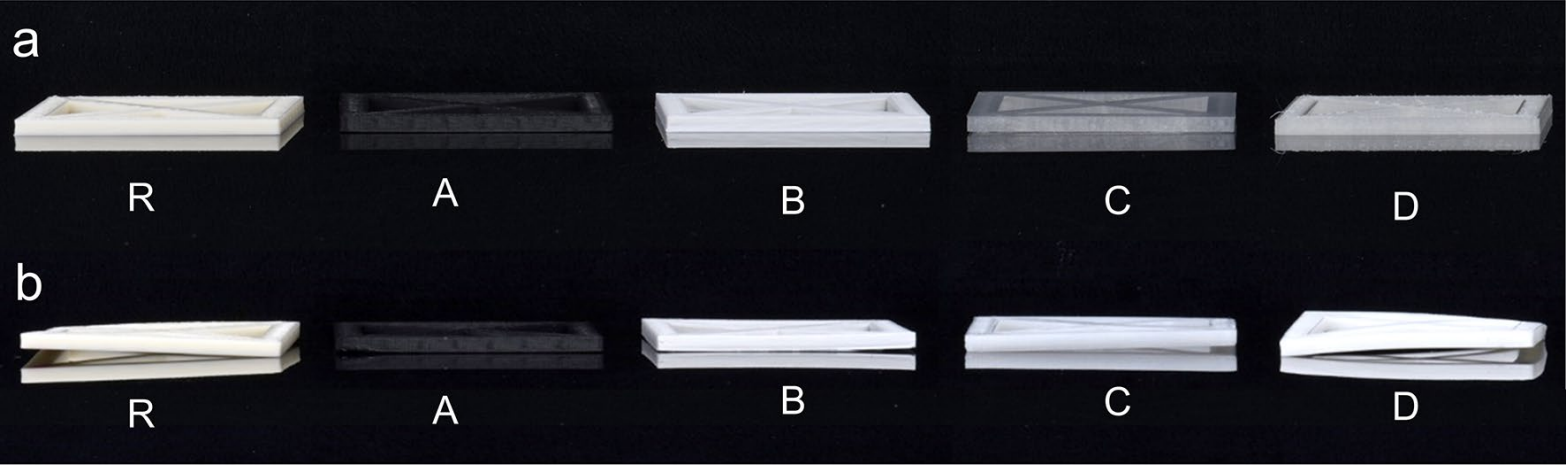
Exemplary visualization of square specimens (R, A–D) after 3D printing (**a**), and after steam sterilization (**b**).

**Figure 4:**
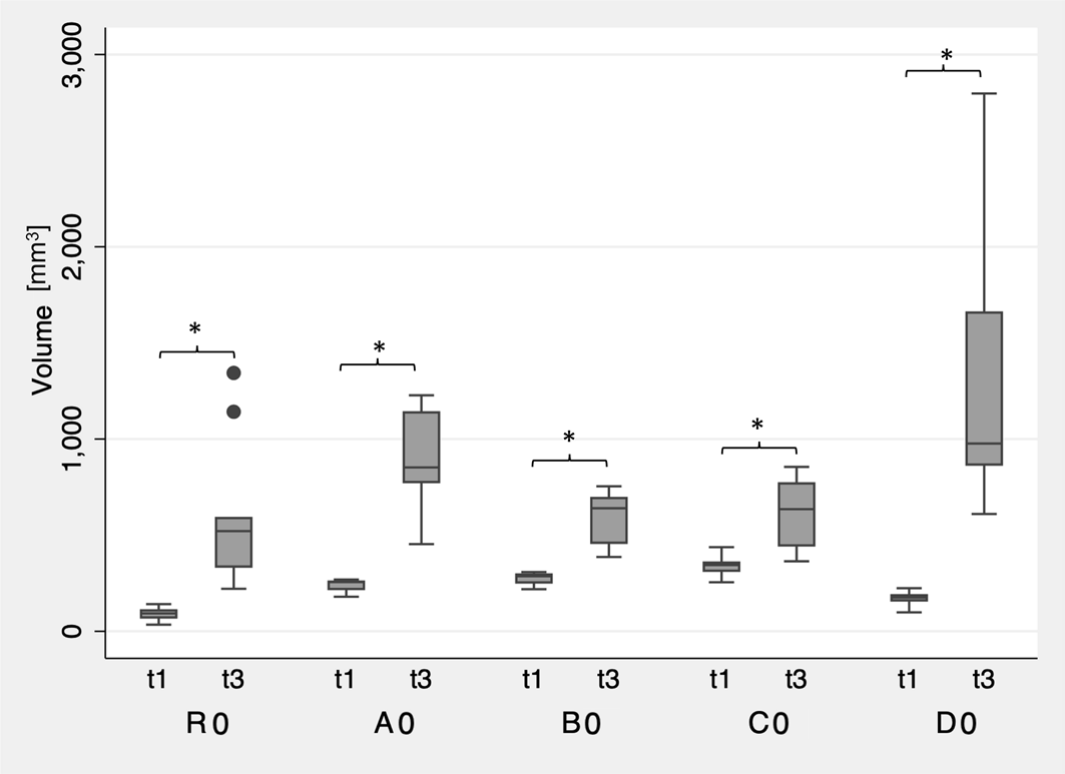
Boxplot of the dimensional changes of the investigated groups (R0, A0–D0,) after 3D printing (t1) and after steam sterilization (t3) compared to the original STL file. Asteriks (*) indicate significances within the groups.

At visual inspection after extrusion-based printing, test specimens of the investigated groups showed only minimal warpage (Fig. 3a). However, the different specimens showed significant differences in warpage after extrusion-based printing (p < 0.05). This outcome can also be attributed to the shrinkage of the specimens after printing. After subsequent steam sterilization at 134 °C, all investigated groups showed a significant increase in warpage (p < 0.01) (Figs. 2b, 3b, 4). Moreover, the standard deviations of all groups increased, and group D0 revealed the largest standard deviation. This group also exhibited the highest increase in warpage due to sterilization and showed significant differences compared to all other groups after steam sterilization (p < 0.01). No further significant differences were observed between the groups after sterilization (p > 0.05). Data of all measured deviations are given in the supplementary material.

**Figure 5:**
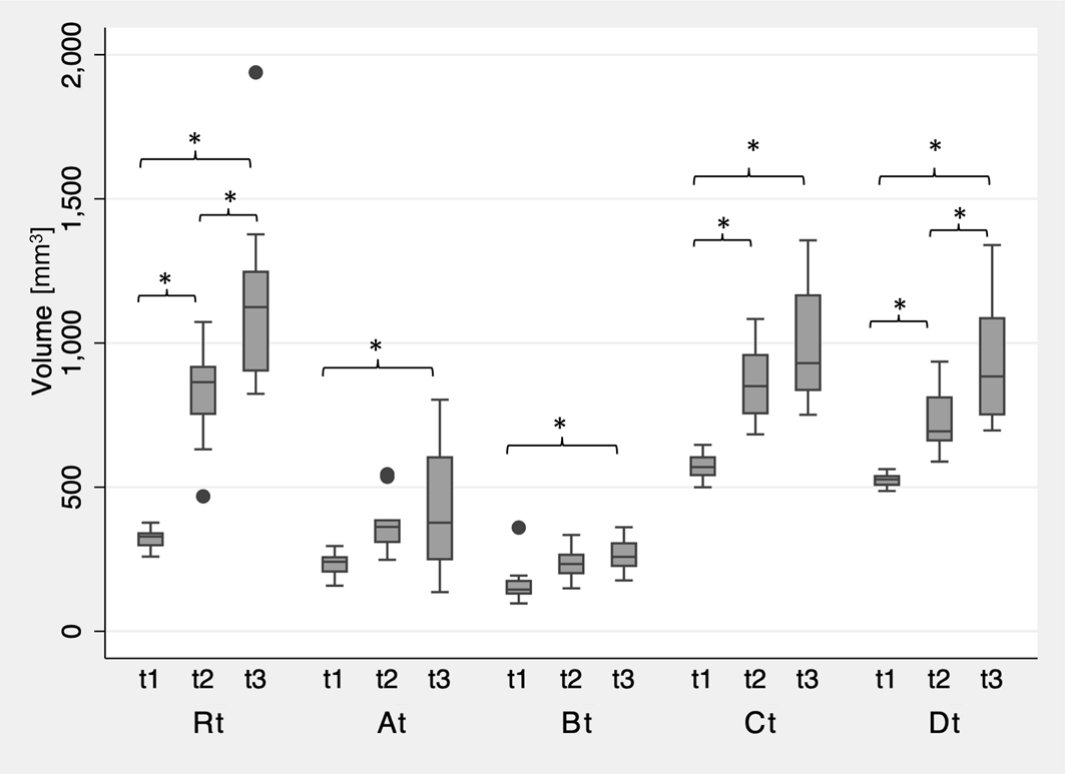
Boxplot of the dimensional changes of the investigated groups (At–Dt, Rt) after 3D printing (t1), annealing (t2), and steam sterilization (t3) compared to the original STL file. Asteriks (*) indicate significances within the groups.

Compared to the groups R, C and D, groups A and B were manufactured with consistent dimensions in a second batch. In particular, group A revealed the lowest difference between both batches (A0: 240 ± 50 mm^3^; At: 236 ± 42 mm^3^). All groups had low standard deviations of the dimensional changes after printing and showed significant differences among themselves (p < 0.05) (Fig. 5). Thermal annealing caused a significant increase in warpage for groups Rt, Ct and Dt (p ≤ 0.01). Groups At and Bt containing fillers and nucleating agents, showed no significant dimensional change due to annealing (At: p = 0.11; Bt: p = 0.07). The subsequent sterilization process did not result in any significant warpage compared to the test specimen geometry after annealing (At: p = 0.67; Bt: p = 0.56). However, in comparison to the dimensions of the test specimens after printing, significant differences were observed for both groups compared to their dimensions after sterilization (At: p = 0.02, Bt: p = 0.01). No significant differences were found between At and Bt (p > 0.05). The sterilization process caused significant warpage of the annealed specimens of the groups Rt and Dt (p < 0.05). In contrast, group Ct showed no significant warpage due to sterilization after preceding annealing (p = 0.2).

### Variation of printing parameters

**Figure 6:**
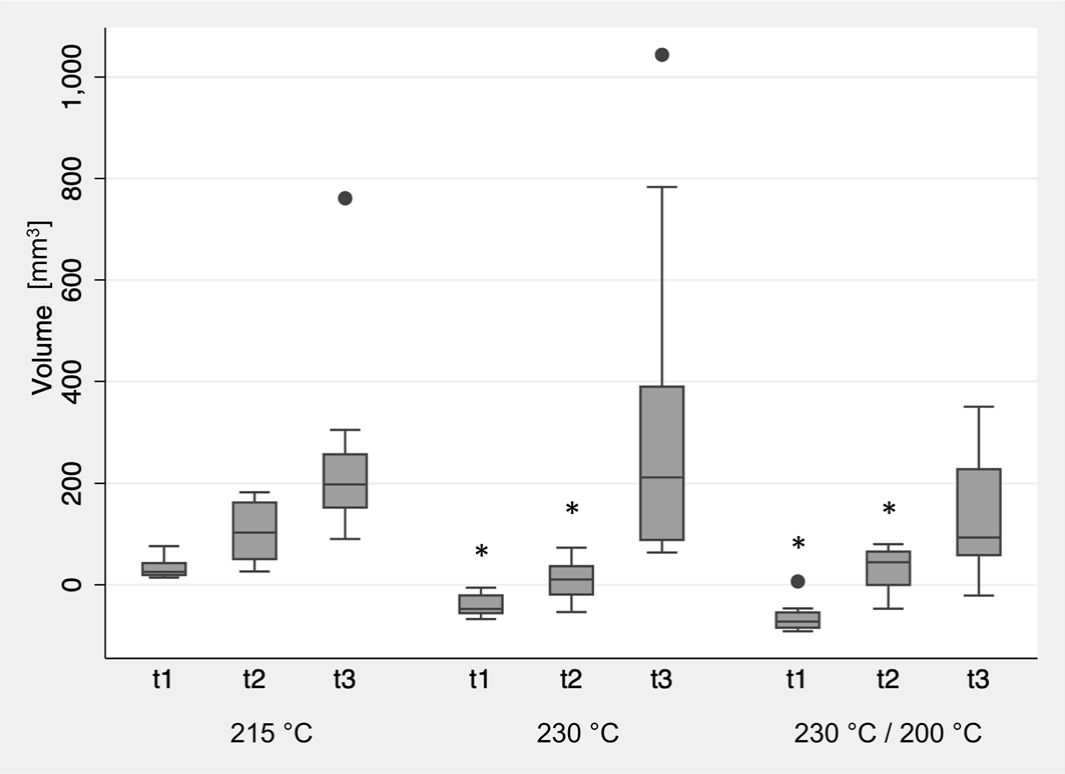
Influence of nozzle temperature on the dimensional changes of group At after 3D printing (t1), annealing (t2), and steam sterilization (t3) compared to the original STL file. Asterisks (*) indicate the significance to standard printing temperature of 215 °C at the respective time point.

Printing of the square specimens (group At) with both a nozzle temperature of 230 °C and with the first layer at 230 °C followed by a subsequent printing temperature of 200 °C resulted in reduced dimensions of the square specimens (p < 0.05 between the groups) (Fig. 6). The sterilization process resulted in significant warpage for all groups, whereas it caused a greater increase in warpage for the group printed with 230 °C (p < 0.05). However, the modified printing temperatures caused no differences between each other at t3 (p > 0.05).

**Figure 7:**
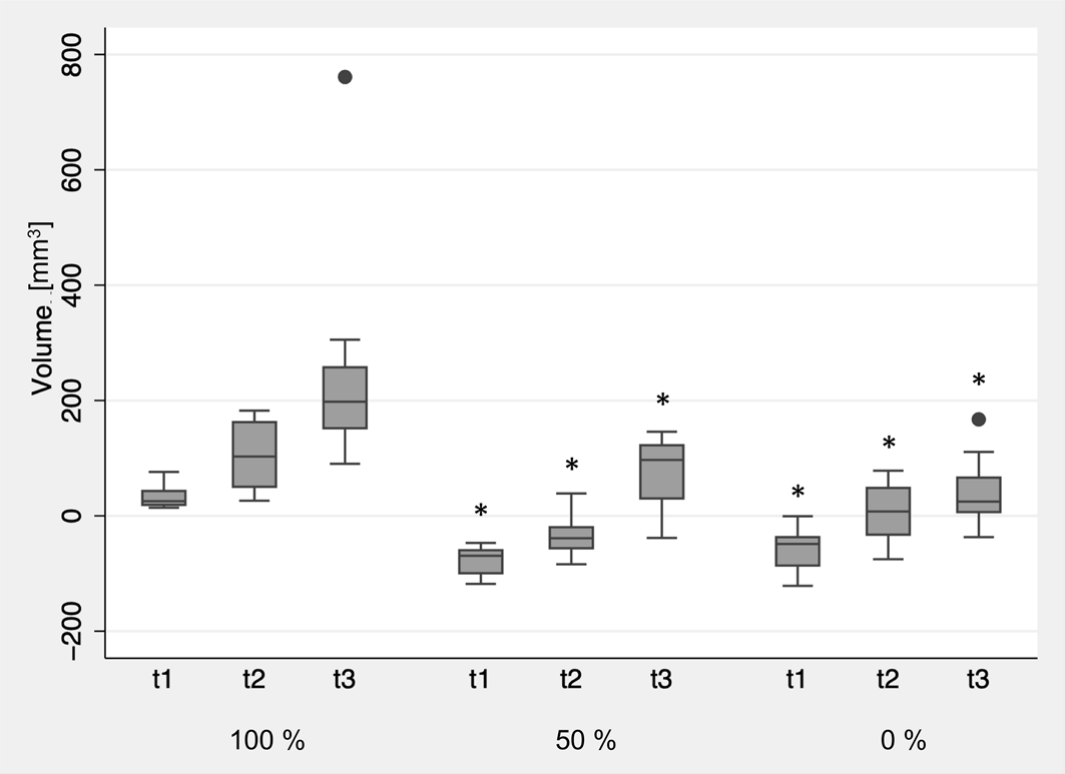
Influence of infill on the dimensional changes of group At after 3D printing (t1), annealing (t2), and steam sterilization (t3) compared to the original STL file. Asterisks (*) indicate significance to standard printing infill of 100% at the respective time point.

Printing of the square specimens with a lower infill caused significantly reduced dimensions of the square specimens compared to the applied standard printing parameter with 100% infill (p < 0.01) (Fig. 7). No significant differences were observed between the two groups manufactured with a modified infill (p = 0.13). Annealing and sterilization resulted in a further dimensional deviation for all investigated groups (t3 and t1; p < 0.01). However, the groups printed with 0% and 50% infill showed lower overall warpage than those printed with 100% infill (p < 0.01). No significant differences were observed between the groups printed with reduced infill (0%; 50%) (p = 0.47).

**Figure 8:**
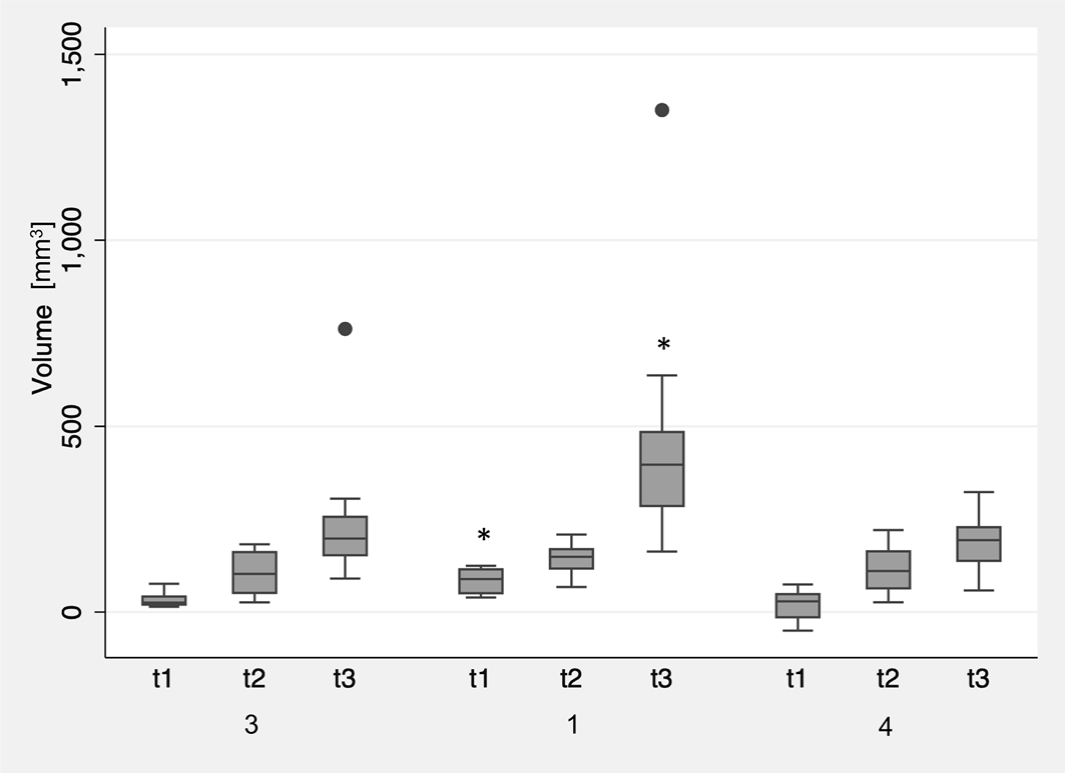
Influence of the number of horizontal and vertical shells on the dimensional changes of group At after 3D printing (t1), annealing (t2), and steam sterilization (t3) compared to the original STL file. Asterisks (*) indicate significance to standard printing parameters with 3 shells at the respective time point.

After printing, no significant differences between the groups printed with 3 and 4 outer shells were observed (p = 0.44) (Fig. 8). However, printing with 1 shell showed an increased deviation (p < 0.01). These specimens exhibited no significant increase in warpage due to annealing (p = 0.8), however, steam sterilization caused significant warpage (t3 to t2: p = 0.01), resulting in the highest dimensional deviation from the original STL file (469 ± 340 mm^3^; p < 0.05 to the other groups). The specimens printed with 4 outer shells showed a significant increase in warpage after annealing and sterilization (p < 0.01). Nevertheless, the overall deviation to the original STL was the lowest compared to the other two groups (t3: 191 ± 75 mm^3^).

### Evaluation of extrusion based printed surgical guides

**Figure 9:**
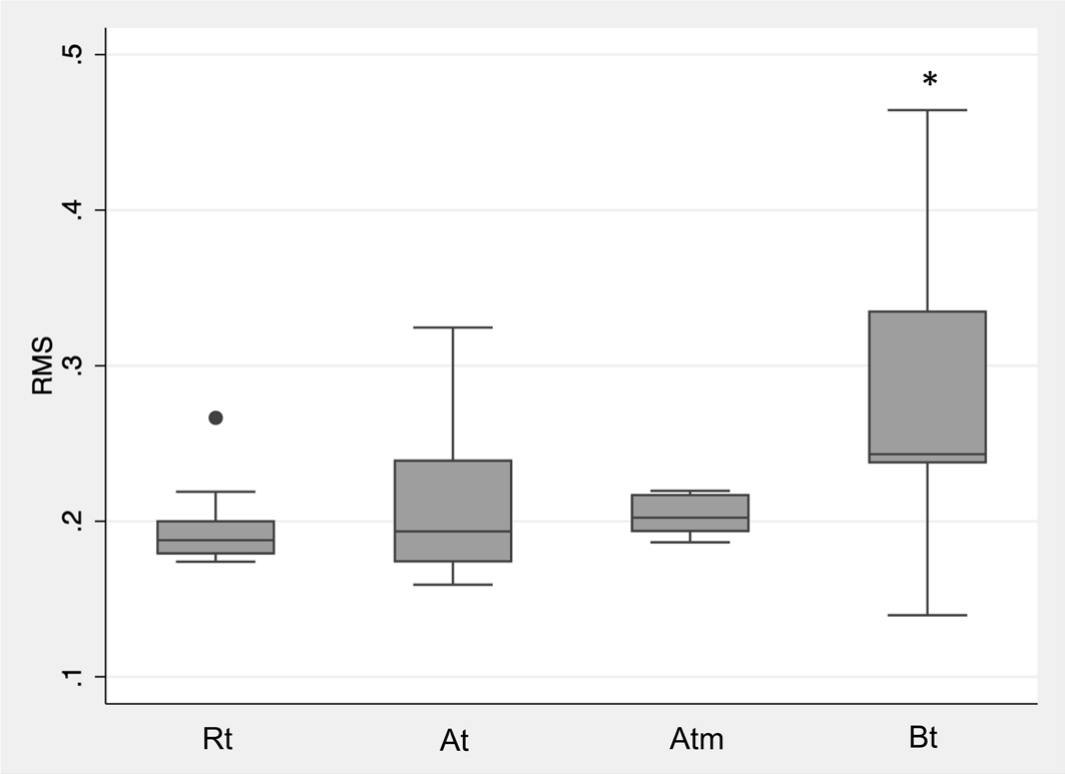
Root Mean Square (RMS) values of extrusion-based surgical guides (Rt, At, Atm, Bt) after thermal annealing and steam sterilization. Bt showed significant differences (*p < 0.05) to the other evaluated groups.

**Figure 10:**
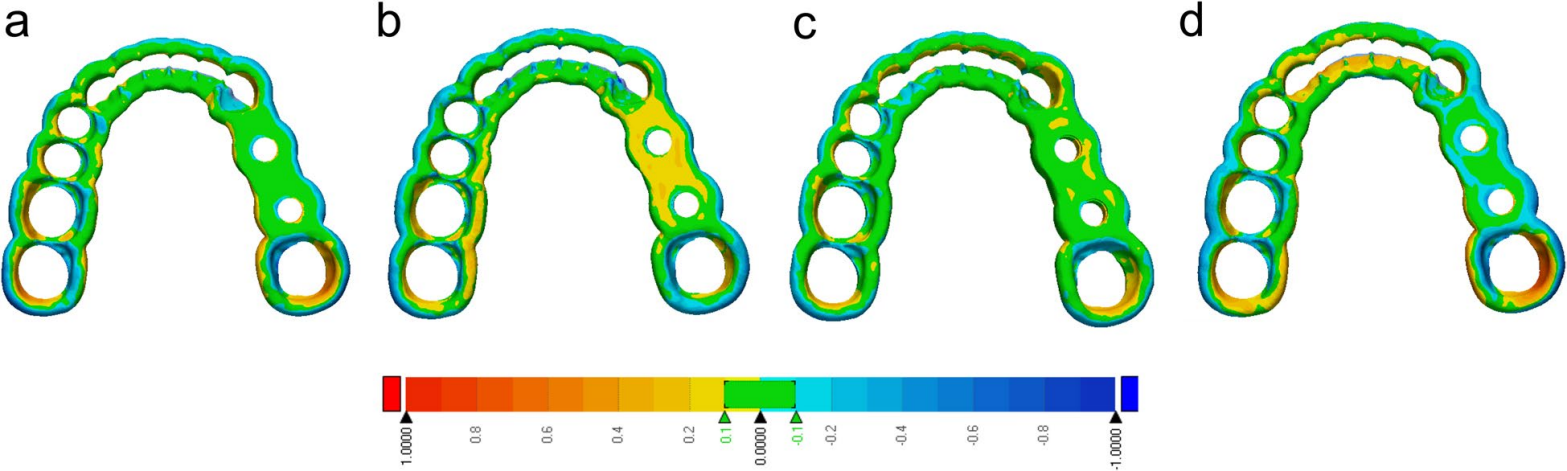
Exemplary visualization of heatmaps indicating the deviation of surgical guides of group Rt (**a**), At (**b**), Atm (**c**), and Bt (**d**) after annealing and steam sterilization. Areas in green show deviations below 0.1 mm.

Since groups At and Bt showed the least deviation from the original STL for the square specimens after annealing and sterilization, these groups were evaluated for the printing of surgical guides. In addition, group At was investigated with modified printing parameters (Atm) based on the variation of printing parameters with the square specimens. Solely group Bt revealed significant differences regarding the RMS value to all other evaluated groups (p = 0.01) (Fig. 9). Exemplary heatmaps of the surgical guides are used to visualize the deviations from the STL (Fig. 10). All groups showed a tendency towards an increased deviation in the region of the last molar, next to the implant region. This tendency was found to be more distinct for group Bt. The heat maps indicate that most of the surface of the surgical guides showed less than 0.1 mm deviation from the STL after annealing and steam sterilization (Fig. 10, green areas).

### TGA and DSC analyses

**Figure 11:**
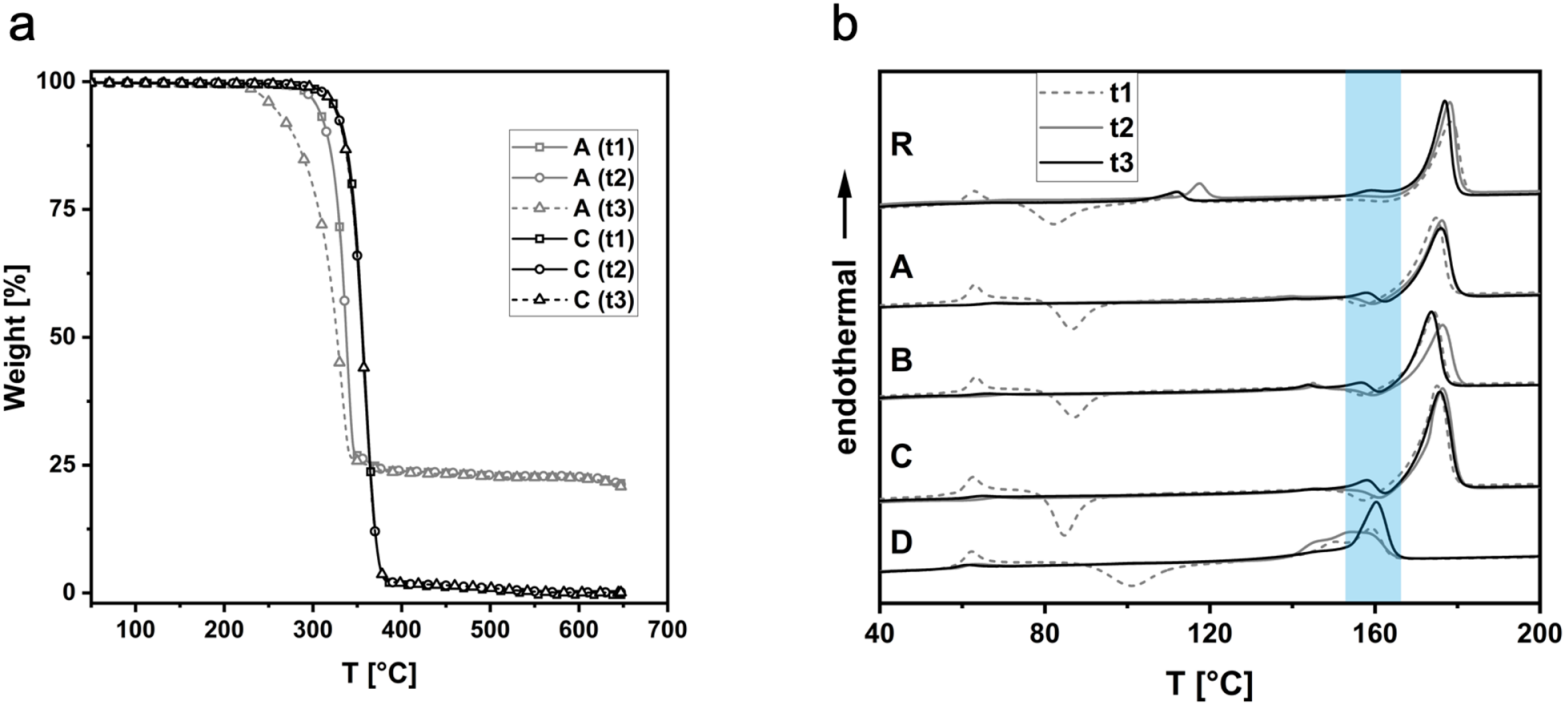
TGA of samples of group A and C (**a**). The residue at 650 °C indicates that A contains inorganic filler material. While C exhibits similar onset temperatures after printing (t1), annealing (t2), and sterilizsation (t3), A shows a significantly reduced onset temperature after t3 indicating a preceeding degradation or aging of the material; DSC first heating cycle (10 K min^−1^) of all samples after t1, t2, and t3 (**b**) with a blue bar marking the melting area of crystalline PLA.

TGA analysis showed a material residue for groups A, B, and R at 650 °C (Table 2). This indicates a mineral filler content that was lowest for R (14wt%) and highest for group B (32wt%). Group C and D showed no residue at 650 °C, indicating no inorganic filler content. Annealing (t2) did not affect the onset temperature of any of the samples. However, samples with inorganic fillers (A, B, R) exhibited a reduced onset temperature after sterilization (t3), which indicates a preceding degradation or aging of the materials. This was not observed for the filler-free samples C and D, as exemplarily illustrated in Fig. 11.

**Table 2:** TGA analyses of the evaluated groups.

Group	Time	TGA		DSC 1. Heating Cycle			
		Onset-temp. [°C)	Residue at 650 °C [%]	Tm1 [°C]	ΔH(Tm1)^a^ [J g^−1^]	Tm2 [°C]	ΔH(Tm2)^a^ [J g^−1^]
R	t1	334	14.7	n.a	n.a	178	47
	t2	334	13.5	n.a	n.a	178	46
	t3	325	14.5	159	3	177	45
A	t1	322	21.4	n.a	n.a	175	44
	t2	322	21.2	n.a	n.a	176	44
	t3	300	20.8	159	3	176	43
B	t1	312	31.7	n.a	n.a	174	47
	t2	311	32.4	n.a	n.a	176	46
	t3	293	32.2	159	4	174	46
C	t1	340	0	n.a	n.a	175	47
	t2	339	0	n.a	n.a	176	46
	t3	339	0	159	6	176	46
D	t1	341	0	159	16	n.a	n.a
	t2	340	0	154	18	n.a	n.a
	t3	340	0	160	29	n.a	n.a

^a^ΔH corrected for filler content according to TGA residue at 650 °C.

In DSC analyses, all groups showed a glass transition temperature (Tg) of approximately 60 °C, indicating PLA as a basis material (Table 2). In addition, the melting point at approximately 170 °C indicates that all groups except for D also contain polyhydroxybutyrate (PHB). Considering the different filler content, the integral of the PHB melting peak and thus the crystallinity of the materials is comparable for all samples. However, after steam sterilization (t3), all samples exhibit an additional melting point at around 160 °C, which is characteristic for PLA. This outcome indicates that the elevated temperatures during steam sterilization induce a crystallization of the PLA, which is otherwise amorphous under 3D printing conditions due to its slow crystallization rate. The PLA melting peak is particularly pronounced in the filler-free samples C and D.

## Discussion

Implementing FFF in the medical sector by using thermoplastic biopolymers seems promising: Devices such as surgical guides or medical instruments for single use can be custom made and the simplified manufacturing technique allows convenient handling for the practitioner^21^. Furthermore, the use of biopolymers can have a positive impact on the environment if they are produced from renewable feedstocks. PLA, PBAT, and PHA are furthermore biodegradable under specific conditions and can thus counteract uncontrolled littering and landfill^22^. On the other hand, FFF still faces challenges when applied in the professional medical sector: The printed objects must prove stable dimensions and should be autoclavable with a convenient protocol for the use in a sterile environment. Therefore, this study evaluated the dimensional accuracy of different biocopolyester blends after extrusion-based printing, annealing, and steam sterilization.

A square specimen, which is prone to warpage, was selected as the test specimen. In this design, the contraction of the constituting straight sections is enhanced by the longitudinal orientation of the strands. This leads to increased pulling forces upon the corners of the warpage specimens. The design was already used in previous studies evaluating the warpage of FFF printable polypropylene^20,23^, with the side length shortened in the present study to approximate the size of a surgical guide. In a previous study, one corner was fixed with a specific force and the height of the corner on the opposite side was measured, from which the warpage was quantified^20^. The present study determined the warpage after digitization using a 3D profilometer to achieve higher accuracy in the measurement. The enclosed volume below the squares was determined, from which the volume of the original STL dataset was subtracted.

All of the evaluated materials were biocopolyester blends containing PLA, PBAT, and PHA. It was observed that the composition of the materials, and in particular the proportion of fillers, nucleating agents, and melt stabilizers significantly impacted the accuracy of the specimens after printing. Although no visual warpage was noticeable, significant differences were observed between the groups, printed with the same standard parameters. Material D, which was based on a different basis composition compared to materials A–C, showed the highest warpage after sterilization.

Thermal annealing as a post-fabrication method is often used to improve the mechanical properties of extrusion-based printed parts. Commonly, annealing is performed approximately 20 °C below the polymer melt temperature, where amorphous and poorly organized crystalline regions can reorganize^24^. This often leads to a reduction of internal stress^25^, which is conducted with a higher thermal and mechanical stability^26^. Furthermore, annealing can reduce the influence of fluctuating printing conditions on the dimensional accuracy^20^. At significantly lower temperatures, the annealing time has to be prolonged, which results in limitations for a practical application. This thermal post-processing of the printed square specimens revealed different effects on the dimensional accuracy of the test specimens. Materials A and B, which contained a higher amount of mineral fillers (21–32 wt%) as well as nucleating agents, showed no significant dimensional change due to annealing. The subsequent steam sterilization did not lead to significant warpage. Nevertheless, the small warpage of both processing steps added up to significant warpage of the specimens. However, the overall deviation was significantly lower compared to those sterilized without preceding annealing.

The results that annealing in particular did not lead to significantly increased warpage of the test specimens in groups A and B can be attributed mainly to mineral fillers. Nucleating agents increase the nucleation rate during the crystallization of polymers and lead to faster crystallization of the melt. This is achieved by reducing the surface energy barrier to nucleation, allowing polymers to crystallize at higher temperatures^27^. An influence of the nucleating agent on the total crystallinity of the polymer portion of the samples was not detected based on DSC analyses. However, a reduction of the size of the crystallites and an associated lower tendency to warp is possible due to the addition of the nucleating agent. This is the subject of further investigations. Material R with a lower filler content showed high warpage after annealing, indicating that a higher filler content in combination with nucleating agents could be beneficial for reduced warpage during annealing. Since annealing of group R led to a higher increase in warpage compared to the samples subjected to steam sterilization directly after printing suggests no benefit from thermal annealing for group R.

The warpage after sterilization correlates qualitatively with an increasing degree of crystallization of the materials. Due to the increased temperatures of 134 °C during sterilization, crystallization of the PLA occurred. The associated volume shrinkage resulted in warpage. The detected amount of crystalline PLA in sterilized samples was particularly pronounced in the filler-free groups C and D. An increasing amount of mineral fillers counteracted post-crystallization of the PLA and stiffened the material at elevated temperatures, thus counteracting warpage. However, in addition to the positive effect of the mineral fillers on warpage, the fillers also caused accelerated aging or degradation of the material at elevated temperatures during sterilization, which may limit repeated sterilization and use. The significant influence of the filler content on the dimensional stability has also been described for other polymers^28^. Materials with an increased crystalline content, such as polyolefins, benefit from additional fillers^23^. In this case, fillers of small size may lead to a significantly reduced warpage and shrinkage. The filler types, geometries, and sizes also seem to significantly influence the material properties of PLA-based biopolymers^29^. The addition of fillers was found to increase flexural stiffness and improve dimensional stability after solidification of PLA-based filaments^9^. It was shown that calcium carbonate, also contained in materials A and B, can lead to increased thermal stability of PLA^30^, especially in combination with talc^31^. However, the use of fillers might also be accompanied by shortcomings such as reduced tensile strength, brittleness, and an increase in density. Moreover, plastic processing machines are subject to increased wear when using mineral fillers^9^. In terms of full biodegradability, mineral fillers may limit the biodegradability of the evaluated materials. For this reason, using natural fibers, e.g. from pulp, in biodegradable materials is being pursued.

A drawback of PLA is the Vicat softening temperature/melting temperature, which is lower than for thermoplastics with a higher crystalline content. In addition, the processing temperature of PLA is close to its decomposition temperature. However, it was demonstrated that the composition could increase the softening temperature with other biopolymers and additives. In the case of the examined reference material, the Vicat softening temperature was around 160 °C, which was significantly higher than the typical Vicat softening temperature starting at approximately 60 °C for PLA^32^. Nevertheless, compared to polymers with a higher crystalline content, the evaluated biocopolyesters were more sensitive to increased temperatures, e. g. during steam sterilization^33^. In the present study, a steam sterilization protocol with a temperature of 134 °C was selected, representing a frequently applied protocol in dental offices. Similar studies investigating the capability to sterilize various 3D printable materials often used solely a temperature of 121 °C^34,35^. In previous studies, the reference material R was found to have acceptable dimensional stability under steam sterilization at 121 °C^17,36^. Thus, it can be assumed that sterilizing the printed biocopolyester specimens at a temperature of 121 °C would have amounted to a reduced warpage, whereas H_2_O_2_ plasma sterilization could represent another mild alternative. Disadvantageous is the limited availability and the increased costs associated with the sterilization process, which would contradict the aim of a low-budget workflow^37^.

Besides the influence of the material composition (fillers, nucleating agents, melt stabilizers) on the dimensional accuracy of FFF printed parts, the printing parameters and further ambient factors might influence the dimensional accuracy. The present study revealed significant differences of the nozzle temperature, outer shells, and infill on the dimensional accuracy. A reduced number of outer shells resulted in a lower stability of the printed parts, which was particularly evident in increased warpage during sterilization. An increased number of shells was also shown to be beneficial for the mechanical strengths of the samples demonstrated in a study by Dong et al. using PLA^38^. Although the present study revealed a reduced deviation of printed samples at 230 °C compared to the original file, further research is necessary to evaluate the influence of high nozzle temperatures on the mechanical parameters such as strength and stiffness^39^. Many drawbacks might be corrected adapting the various printing parameters, which often requires a trial and error approach^9^. However, even with the applied same parameters, further factors might influence the outcomes, especially when a low-budget workflow is aspired. Since this study aimed to evaluate a workflow that was as affordable and simple as possible, a low-cost desktop extrusion-based printer without further equipment was used. Considering that the printer has no chamber, ambient conditions such as the room temperature and humidity may have influenced the printing quality^8^. Meticulous care was taken to ensure that the filaments were stored in a cool and dry place. Nevertheless, even slight differences in the humidity of the filaments might have contributed to differences in dimensional accuracy between the two cohorts printed with the same printing parameters.

The geometry of the printed object also influences the dimensional printability. While the square specimens tend to warpage and show significant differences, the surgical guides showed fewer differences between the groups. Only group B showed a significantly increased warpage to the other studied groups after annealing and sterilization. The heatmaps revealed that even after steam sterilization, most regions only showed deviations of up to 0.2 mm. In a preclinical study, placed implants with printed surgical guides made of material R showed no significant differences from SLA printed surgical guides^17^. The result that the surgical guides showed less distortion than the squares can be attributed to the fact that they are thicker and thus stiffer than the squares. Additional layers of molten polymer also shrink resulting in less distortion because the stiff areas are strong enough to resist deformation. The last strand deposited must inherently relieve the stress. The fact that the highest warpage of the surgical guides occurred in the molar region is comparable to the warpage tendency of the squares, where increased warpage occurred at the end of the longest printed axis when the shrinkage of the polymer was parallel to it^20^. Nevertheless, it must be determined for the respective application whether a slight distortion has an influence on the respective clinical application. For surgical guides made of these novel biocopolyester blends, the implant position after implant placement with these surgical guides should be compared to the planned position in vitro^17^. Furthermore, the biocompatibility of the evaluated biocopolmers must be tested. A recent study showed that the applied reference material had no cytotoxic effects on oral cells and may be superior in biocompatibility to a commonly used photopolymer for surgical guides^40^. It is reported in different studies that vat polymerization of light-curing resins can lead to cytotoxic effects^41,42^. The promising results regarding the biocompatibility of material R suggest similar outcomes for the other extrusion-based materials, which need to be assessed in further investigations.

## Conclusions

Printed square test specimens of the investigated biocopolyester blends, which tend to warp at elevated temperatures, are thus not dimensionally stable when steam sterilized at 134 °C. This was accompanied by significant crystallization of the samples at elevated sterilization temperatures. However, the material modification with mineral fillers combined with thermal annealing showed a significantly reduced warpage after annealing and steam sterilization. In addition, the filler-containing material modifications exhibited accelerated aging or degradation of the polymer at steam sterilization conditions. The evaluated modifications in combination with thermal annealing represent approaches for the production of thermally stabilized biopolymers that are also biodegradable under certain conditions. Printing of surgical guides, intended for the insertion of oral implants, resulted in decreased warpage between the groups after annealing and steam sterilization.

## Supplementary Information

Supplementary Information.

## Data Availability

All data generated or analyzed during this study are included in this published article.
