# Supplementary material for: Tailoring the composition of biocopolyester blends for dimensionally accurate extrusion-based printing, annealing and steam sterilization
Source: Sci Rep. 2022 Nov 25;12:20341. doi: 10.1038/s41598-022-24991-z (PMC9700831; doi:10.1038/s41598-022-24991-z)
Supplement: Supplementary file 1 — Supplementary Information. [file 41598_2022_24991_MOESM1_ESM.docx]

**Supplementery Material**

**Appendix 1:** Data of groups R0-D0 at t1 and t3 (Figure 4).

| Group | Time point | N | Mean [mm^3^] | Standard Deviation [mm^3^] |
| --- | --- | --- | --- | --- |
| R0 | t1 | 10 | 91.9535 | 31.51437 |
| A0 | t1 | 10 | 239.6352 | 34.53414 |
| B0 | t1 | 10 | 274.12 | 30.84259 |
| C0 | t1 | 10 | 339.1907 | 48.41544 |
| D0 | t1 | 10 | 171.6067 | 36.99114 |
| R0 | t3 | 10 | 496.1545 | 364.8992 |
| A0 | t3 | 10 | 659.1771 | 235.8461 |
| B0 | t3 | 10 | 317.505 | 129.4002 |
| C0 | t3 | 10 | 285.3626 | 207.5868 |
| D0 | t3 | 10 | 1170.851 | 727.8688 |

**Appendix 2:** Data of groups Rt-Dt at t1, t2, and t3 (Figure 5).

| Group | Time point | N | Mean [mm^3^] | Standard Deviation [mm^3^] |
| --- | --- | --- | --- | --- |
| Rt | t1 | 10 | 325.6785 | 36.39799 |
| At | t1 | 10 | 236.4439 | 42.40946 |
| Bt | t1 | 10 | 165.7381 | 74.30911 |
| Ct | t1 | 10 | 572.9132 | 46.4935 |
| Dt | t1 | 10 | 525.2047 | 26.76346 |
| Rt | t2 | 10 | 496.4936 | 165.4886 |
| At | t2 | 10 | 132.9139 | 77.53991 |
| Bt | t2 | 10 | 67.4437 | 80.27962 |
| Ct | t2 | 10 | 289.1199 | 129.8058 |
| Dt | t2 | 10 | 210.8622 | 107.6754 |
| Rt | t3 | 10 | 347.575 | 253.7554 |
| At | t3 | 10 | 55.178 | 140.5853 |
| Bt | t3 | 10 | 30.4005 | 22.73398 |
| Ct | t3 | 10 | 119.8742 | 102.1892 |
| Dt | t3 | 10 | 205.0179 | 193.0874 |

**Appendix 3:** Data regarding the evaluation of different printin parameters (printing temperature) (Figure 6).

| Group | Time point | N | Mean [mm^3^] | Standard Deviation [mm^3^] |
| --- | --- | --- | --- | --- |
| 215 °C | t1 | 10 | 32.5501 | 19.97142 |
| 200 °C | t1 | 10 | -40.1399 | 21.66558 |
| 230 °C /200 °C | t1 | 10 | -64.3461 | 29.06335 |
| 215 °C | t2 | 10 | 70.9522 | 55.47822 |
| 200 °C | t2 | 10 | 50.591 | 43.9219 |
| 230 °C /200 °C | t2 | 10 | 96.9099 | 46.8096 |
| 215 °C | t3 | 10 | 148.9721 | 201.7687 |
| 200 °C | t3 | 10 | 317.5077 | 353.8749 |
| 230 °C /200 °C | t3 | 10 | 92.1806 | 103.1094 |

**Appendix 4:** Data regarding the evaluation of different printin parameters (infill) (Figure 7).

| Group | Time point | N | Mean [mm^3^] | Standard Deviation [mm^3^] |
| --- | --- | --- | --- | --- |
| 100 % | t1 | 10 | 32.5501 | 19.97142 |
| 50 % | t1 | 10 | -78.1506 | 24.12034 |
| 0 % | t1 | 10 | -58.8301 | 36.15437 |
| 100 % | t2 | 10 | 70.9522 | 55.47822 |
| 50 % | t2 | 10 | 43.6985 | 32.68681 |
| 0 % | t2 | 10 | 61.9666 | 51.82515 |
| 100 % | t3 | 10 | 148.9721 | 201.7687 |
| 50 % | t3 | 10 | 114.4112 | 57.6789 |
| 0 % | t3 | 10 | 37.3194 | 78.36243 |

**Appendix 5:** Data regarding the evaluation of different printin parameters (outer shells) (Figure 8).

| Group | Time point | N | Mean [mm^3^] | Standard Deviation [mm^3^] |
| --- | --- | --- | --- | --- |
| 3 | t1 | 10 | 32.5501 | 19.97142 |
| 1 | t1 | 10 | 86.4978 | 34.11229 |
| 4 | t1 | 10 | 20.8906 | 41.85281 |
| 3 | t2 | 10 | 70.9522 | 55.47822 |
| 1 | t2 | 10 | 60.004 | 40.61707 |
| 4 | t2 | 10 | 95.123 | 69.07045 |
| 3 | t3 | 10 | 148.9721 | 201.7687 |
| 1 | t3 | 10 | 322.8072 | 339.634 |
| 4 | t3 | 10 | 75.3458 | 45.86414 |

**Appendix 6:** Data (RMS-Values) of the evaluated groups (Figure 9).

| Group | Time point | N | Mean | Standard Deviation |
| --- | --- | --- | --- | --- |
| Rt | t3 | 10 | 0.196 | 0.02811053 |
| At | t3 | 10 | 0.214 | 0.05648996 |
| Atm | t3 | 10 | 0.2038 | 0.0130979 |
| Bt | t3 | 10 | 0.2841 | 0.0972 |
